# A multi-view TSK fuzzy system with deformable Gaussian membership functions and rule-level attention for classification

**DOI:** 10.1371/journal.pone.0348610

**Published:** 2026-05-11

**Authors:** Zhiqi Huang, Yizhang Jiang, Kaijian Xia

**Affiliations:** 1 School of Artificial Intelligence and Computer Science, Jiangnan University, Wuxi, Jiangsu, China; 2 Engineering Research Center of the Ministry of Education for Intelligent Technology and Healthcare, Jiangnan University, P.R. China; 3 Changshu Key Laboratory of Medical Affiliated Intelligence and Big Data, Suzhou, Jiangsu, China; 4 Center of Intelligent Medical Technology Research, Changshu Hospital Affiliated to Soochow University, Suzhou, Jiangsu, China; University of Bonab, IRAN, ISLAMIC REPUBLIC OF

## Abstract

This study presents a novel multi-view TSK fuzzy system that integrates deformable Gaussian membership functions with a rule-level attention mechanism (MDA-TSK-FS), aiming to improve the modeling capacity and flexibility of fuzzy systems in high-dimensional and complex classification tasks. In the antecedent part, learnable deformation offsets are introduced, enabling the membership function centers of each fuzzy rule to dynamically adjust according to data characteristics. This design enhances the adaptability of rules to the input space. Furthermore, a multi-head attention mechanism at the rule level is incorporated to adaptively allocate rule weights based on sample-specific information, thereby enabling dynamic modeling of rule importance and optimized rule selection. Extensive experiments on five public multi-view datasets, including Caltech7, Handwritten, Dermatology, Forest, and EEG, demonstrate that the proposed model consistently achieves superior performance, reaching classification accuracies of 94.38%, 98.62%, 98.58%, 88.57%, and 69.75%, respectively, and outperforming strong baselines. Ablation studies further verify the effectiveness of the two core components: the deformable antecedent structure and the rule-level attention mechanism, which individually improved EEG classification accuracy by approximately 7% and 6%, and jointly by 9.25% compared to the baseline. Notably, the model exhibits superior generalization and interpretability, particularly when processing multi-source heterogeneous data. These findings indicate that the proposed approach provides a new modeling paradigm for multi-view fuzzy inference, offering both theoretical contributions and practical application potential.

## 1. Introduction

With the increasing availability of diverse feature representations, single-view learning often fails to capture the full characteristics of complex samples. Multi-view learning, in contrast, provides complementary representations of the same object from different perspectives or feature subsets, enabling joint modeling that strengthens both representational capacity and discriminative power. For example, in image recognition, combining shape, texture, and color features from the same image can improve classification accuracy; in medical diagnosis, integrating multiple biomarkers or imaging sequences of the same patient enhances disease characterization; in bioinformatics, combining gene expression and DNA methylation profiles of the same sample offers deeper insights into molecular mechanisms. By exploiting the consistency and complementarity among views, multi-view learning has become a critical approach for effectively leveraging high-dimensional and heterogeneous data.

In recent years, multi-view learning, which enables joint modeling with multiple feature representations, has received significant attention in both academic research and practical applications [1]. By jointly modeling multiple complementary views, it can effectively improve classification accuracy and robustness across various domains. These advances motivate the development of more flexible and interpretable multi-view inference methods. This study specifically focuses on addressing the challenges of high-dimensional and heterogeneous datasets.

Takagi–Sugeno–Kang (TSK) fuzzy system, characterized by its strong interpretability and rule-driven nature, constitutes an ideal framework for multi-view fusion [2]. TSK models partition the input space through fuzzy antecedent rules and generate outputs using consequent functions, thereby facilitating a rule-based decision-making process. These features make TSK models particularly suitable for applications that demand interpretability and transparency. Nevertheless, despite their advantages, the application of TSK fuzzy systems in multi-view learning encounters several critical challenges:

1. Rule explosion and computational complexity. In conventional TSK models, antecedents are typically constructed using fixed-form Gaussian membership functions (GMFs) to partition the input space. While effective in low-dimensional data, such methods lead to an exponential increase in the number of rules in high-dimensional and multi-view settings, a phenomenon often referred to as the “curse of dimensionality” [3]. This exponential growth not only results in complex model structures and high computational costs but also severely limits efficiency in both training and inference.
2. Limited expressiveness of fixed membership functions. Traditional GMFs possess a fixed structure, with centers and variances that are difficult to adapt to complex data distributions. In multi-view scenarios, heterogeneous and divergent distributions frequently exist across views. Consequently, fixed GMFs often fail to accommodate such diversity. This limitation weakens representational capacity and degrades both classification accuracy and generalization ability [4].
3. Redundant rule activation and inflexible fusion. In practical applications, multi-view data often contain redundant information, such as highly correlated features across views. Without proper differentiation, these redundancies lead to excessive rule activation, diminishing the discriminative capability of the system [5]. Furthermore, conventional TSK models compute rule activations in a fixed manner, which prevents the dynamic adjustment of rule importance according to data characteristics, further hindering model performance and robustness.

To address the aforementioned issues, three primary motivations are identified. First, existing TSK models require extensions beyond fixed-form membership functions to better adapt to the complex distributions of heterogeneous multi-source data. Second, mechanisms are needed to effectively distinguish between informative and redundant rules and dynamically adjust their weights, thereby enhancing discriminability and robustness. Finally, it is crucial to achieve efficient computation and accurate prediction while maintaining interpretability, which is a prerequisite for practical deployment of multi-view fuzzy systems. To this end, a novel multi-view TSK fuzzy system with deformable Gaussian membership functions and an attention mechanism (MDA-TSK-FS) is developed in this study.

The major contributions of this work can be summarized as follows:

1. Deformable Gaussian membership functions (Deformable GMFs). Traditional GMFs are extended by introducing learnable center offsets and shape-adjustment parameters, enabling dynamic adaptation to data distributions. This enhancement significantly improves the capacity to represent complex data structures and alleviates the limitations of fixed GMFs in high-dimensional multi-view settings [6].
2. Rule-level attention mechanism. A novel attention-based rule weighting strategy is proposed, which adaptively adjusts the importance of rules during the activation process. This mechanism suppresses redundant rules, thereby improving discriminability and robustness, and facilitates more effective utilization of complementary information across multiple views [7].
3. Integration into the MDA-TSK-FS framework. By combining deformable GMFs with the rule-level attention mechanism, a new multi-view TSK fuzzy system is constructed. The proposed framework maintains interpretability while significantly enhancing adaptability and modeling capacity for heterogeneous data. Comparative experiments demonstrate that MDA-TSK-FS outperforms traditional GMF-based models and deformable variants without attention in terms of classification accuracy, training efficiency, and generalization ability.

This dual innovation mitigates the dimensionality explosion and rule redundancy problems inherent in traditional multi-view TSK frameworks, enabling efficient and flexible multi-view feature fusion. Extensive experiments on multiple public datasets demonstrate that the proposed model consistently outperforms classical Gaussian membership function (GMF) systems and deformable models without attention, while retaining strong interpretability [8].

The remainder of this paper is organized as follows: Section 2 reviews related work on multi-view learning, TSK fuzzy systems, and recent advances involving deformable membership functions and attention mechanisms. Section 3 presents the proposed model architecture and fusion strategy in detail. Section 4 reports experimental results, comparative evaluations, and ablation studies. Section 5 concludes the paper and outlines future research directions.

## 2. Related work

This section reviews prior research in the following areas: (1) multi-view learning, (2) TSK fuzzy systems and their multi-view extensions, and (3) deformable and attention mechanisms in fuzzy models. Most existing studies employ fixed-structure Gaussian membership functions, lacking mechanisms for dynamic modeling of rule importance and semantic differences among views. To address this gap, we introduce a more flexible and adaptive membership function structure, along with an attention-based rule-weight adjustment strategy, to enhance the expressive power and interpretability of fuzzy systems in multi-view learning scenarios.

### 2.1 Multi-view learning

Multi-view learning aims to exploit complementary information from different modalities or feature spaces to improve generalization and robustness. Canonical Correlation Analysis (CCA) is a classical linear multi-view learning method [9], whose core idea is to find projection directions that maximize the correlation between two views. Given feature matrices from two views $\text{X}\in R^{n\times d_{\text{1}}}$ and $Y\in R^{n\times d_{\text{2}}}$, CCA solves the following optimization problem to obtain the projection directions $W_{x}$ and $W_{y}$:


$$\begin{array}{c} \underset{w_{x},w_{y}}{\text{max}}\;corr\left(X_{W_{x}},Y_{W_{y}}\right)=\frac{\overset{T}{\underset{x}{w}}C_{XY}w_{y}}{\sqrt{\overset{T}{\underset{x}{w}}C_{XX}w_{x}}\cdot \sqrt{\overset{T}{\underset{y}{w}}C_{YY}w_{y}}} \end{array}$$ (1)

Where $C_{XX}$, $C_{YY}$ and $C_{XY}$ denote the covariance and cross-covariance matrices. By maximizing (1), CCA identifies the most correlated subspaces for feature compression and fusion.

For nonlinear relationships, Deep CCA extends CCA by introducing neural networks as feature extractors, learning nonlinear mappings $f_{x}(\cdot )$ and $f_{y}(\cdot )$ before performing correlation maximization. The objective remains similar to CCA:


$$\begin{array}{c} \underset{f_{x},f_{y}}{\text{max}}\;corr\left(f_{x}(X),f_{y}(Y)\right) \end{array}$$ (2)

Deep CCA and its variants (e.g., DMCCA) have become popular for multi-view representation learning [10]. While CCA-based methods are theoretically appealing for learning shared representations, they lack explicit modeling of rule importance and semantic discrepancies between views, making them less interpretable in the context of fuzzy systems. Fine-grained multi-view fusion under fuzzy frameworks thus remains a challenge.

### 2.2 TSK fuzzy systems and multi-view extensions

TSK fuzzy systems are widely used in classification and regression due to their interpretable rule-based structure [8]. In traditional TSK systems, antecedents are defined by fixed-form membership functions, while consequents are typically linear functions-balancing nonlinear modeling capacity with interpretability.

A standard TSK rule can be expressed as:


$$\begin{array}{c} R^{k}:If\;x_{\text{1}\;}is\overset{k}{\underset{i}{\;A}}and\;x_{\text{2}\;}is\overset{k}{\underset{\text{2}}{\;A}}\ldots then{\;y}^{k}=\sum_{i=\text{1}}^{d}\overset{k}{\underset{i}{a}}x_{i}+b^{k} \end{array}$$ (3)

The Gaussian membership function for antecedent $\overset{k}{\underset{i}{A}}\left(x_{i}\right)$ is:


$$\begin{array}{c} \overset{k}{\underset{i}{\mu }}\left(x_{i}\right)=exp\left(-\frac{{\left(x_{i}-\overset{k}{\underset{i}{c}}\right)}^{\text{2}}}{\text{2}{\left(\overset{k}{\underset{i}{\sigma }}\right)}^{\text{2}}}\right) \end{array}$$ (4)

The firing strength of rule k is:


$$\begin{array}{c} f_{k}(x)=\prod_{i=\text{1}}^{d}\overset{k}{\underset{i}{\mu }}\left(x_{i}\right) \end{array}$$ (5)

The system output is computed as:


$$\begin{array}{c} y(x)=\sum_{k=\text{1}}^{K}\frac{f_{k}(x)}{\sum_{j=\text{1}}^{K}f_{j}(x)}\cdot y^{k}(x) \end{array}$$ (6)

For multi-view problems, some works design view-specific rules or fuse rule activations from different views to enhance integration. However, fixed-shape membership functions limit representational capacity when handling high-dimensional, heterogeneous multi-view data, making it difficult to capture complex intra-view and inter-view feature distributions.

### 2.3 Deformable membership functions

To improve adaptability to complex data distributions, deformable membership functions have recently attracted attention [11]. By introducing learnable deformation parameters—such as center offsets and width adjustments—membership functions can dynamically reshape, leading to more accurate partitioning of the input space and enhanced rule discriminability. Compared to fixed-parameter Gaussians, deformable membership functions offer greater flexibility and expressiveness, and have shown advantages in certain fuzzy and neuro-fuzzy models.

The idea draws inspiration from Deformable Convolutional Networks (DCN) in computer vision [12], which introduce learnable spatial offsets to convolution kernels for modeling object deformations. Drawing a direct analogy, just as DCNs break the fixed grid structure to handle irregular object shapes in the spatial domain, our proposed Deformable GMFs break the fixed distribution constraints of traditional fuzzy sets. By introducing learnable offsets to the membership function centers, the model can dynamically align with heterogeneous and irregular data distributions in the high-dimensional feature space. This capability is analogous to ‘deforming’ the receptive field in vision, allowing the fuzzy system to capture complex intra-class variations that static Gaussian functions fail to model.“ Similarly, in fuzzy systems, deformable Gaussians allow the rule boundaries to adapt dynamically to data distributions, rather than remaining static.

Recent research includes deformable Gaussian functions, stretched/twisted membership functions, and other learnable shape-parameterized functions [11]. These methods have demonstrated improved adaptability to heterogeneous, multimodal, and asymmetric data, especially in high-dimensional classification and regression. The deformable Gaussian antecedent proposed in this paper follows this line of research, extending the traditional GMF by adding offset parameters to boost expressiveness and flexibility.

### 2.4 Attention mechanisms in fuzzy systems

Attention mechanisms, a cornerstone of modern deep learning, dynamically adjust the focus of models on specific regions or features based on the input [13]. Incorporating attention into fuzzy systems allows adaptive reweighting of rules or views during activation and fusion, improving both discriminative ability and robustness.

Initially popularized in natural language processing, attention mechanisms have since been widely adopted in computer vision and time-series modeling. In fuzzy systems, attention has been applied to regulate rule importance, enhancing adaptability and data awareness [14]. For example, MIC_FuzzyNet leverages additive attention to selectively enhance certain rule activations, although it does not integrate with antecedent structural improvements [15].

In multi-view fuzzy frameworks, the joint design of attention with deformable membership functions remains largely unexplored. Coordinating these two mechanisms has the potential to improve dynamic rule activation, mitigate redundancy, and fully exploit semantic complementarity across views.

## 3. Methodology

The proposed model is a Multi-view Deformable Gaussian and Attention-based TSK Fuzzy System (MDA-TSK-FS). It takes multi-view features as input and constructs an independent antecedent network for each view to perform fuzzy partitioning.

Within each view, we employ Deformable Gaussian Membership Functions (Deformable GMFs) as the antecedent modeling tool. By introducing learnable offset parameters to dynamically adjust the Gaussian centers, the fuzzy partitioning gains enhanced flexibility and expressive power under complex data distributions.

To further increase the dynamic adaptability of rule selection, the model incorporates an attention mechanism that automatically assigns importance weights to fuzzy rules based on the current input sample. This mechanism effectively regulates the firing strengths of rules, enabling the model to focus on highly discriminative rules and views even in the presence of high-dimensional or redundant features.

The fuzzy rule responses from different views are then integrated in the output layer through a fusion strategy, achieving efficient cross-view information collaboration. Finally, the fused fuzzy inference results are fed into the TSK consequent part, where a linear combination generates the classification output.

The entire training process adopts a cross-entropy loss function, with the AdamW optimizer, and applies an early stopping strategy to alleviate overfitting. Overall, the architecture preserves interpretability while significantly enhancing the model’s representational capacity and classification performance in multi-view scenarios.

### 3.1 Model structure overview

**Fig 1:**
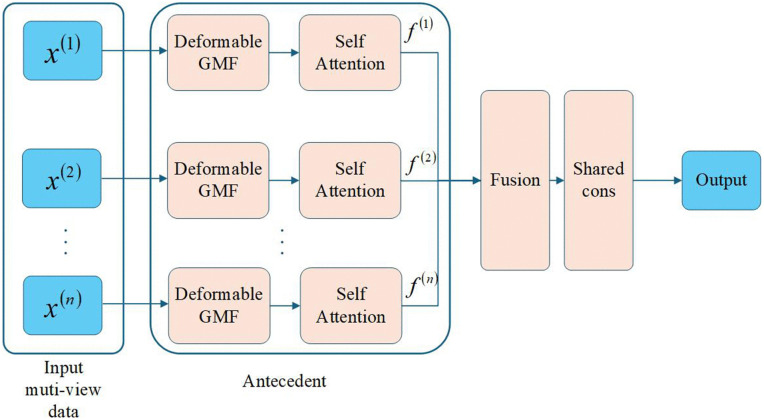
Architecture of the MDA-TSK-FS model.

This study proposes a novel multi-view TSK fuzzy system that integrates deformable Gaussian membership functions with a rule-level attention mechanism for high-dimensional heterogeneous data. As shown in Fig 1, the architecture consists of three modules: a deformable antecedent layer, an attention regulation module, and a TSK consequent layer, deployed as parallel fuzzy subsystems in the multi-view setting.

Each view is processed by an independent deformable Gaussian antecedent network, where learnable offset parameters allow membership function centers to adapt dynamically to data distributions. The resulting rule activations are then refined by a self-attention mechanism that adaptively assigns rule importance based on the current input, improving discriminative focus.

Finally, activations from all views are fused at the rule level to form a global rule response, which is passed to the TSK consequent layer for weighted inference. The end-to-end trainable design preserves interpretability while improving adaptability and classification accuracy.

### 3.2 Deformable Gaussian membership function

In conventional TSK fuzzy systems, the antecedent membership functions are typically fixed-parameter Gaussians(as defined in Eq. (4)). Where $C_{i}$ and $\;\sigma_{i}$ denote the center and width parameters, respectively. While simple, this static formulation has limited adaptability in high-dimensional or complex data distributions, often resulting in imprecise fuzzy boundaries and reduced rule activation effectiveness.

To enhance flexibility and data adaptivity, we introduce the Deformable Gaussian Membership Function, which augments the center with a learnable offset$\Delta c_{ij}$:


$$\begin{array}{c} \mu_{ij}(x)=exp\left(-\frac{{\left(x_{i}-\left(c_{ij}+\Delta c_{ij}\right)\right)}^{\text{2}}}{\text{2}\overset{\text{2}}{\underset{ij}{\sigma }}}\right) \end{array}$$ (7)

Where $c_{ij}\in R$ is the initial center, $\Delta c_{ij}\in R$ is a trainable deformation term, and $\sigma_{ij}$ is the standard deviation.

This formulation enables a deformable fuzzy partition, allowing membership functions to dynamically adjust their centers during training in response to data characteristics. Functionally, this can be viewed as a weak attention mechanism at the antecedent level, improving boundary precision and capturing local variations. The approach retains the Gaussian form, thus preserving interpretability, while substantially improving nonlinear modeling capacity—particularly in scenarios with complex class boundaries or localized perturbations.

### 3.3 Attention mechanism for rule activation

In multi-rule fuzzy systems, the degree to which an input sample activates each rule can vary substantially.

Traditional TSK models compute activations directly from membership functions and apply a simple normalization, ignoring deeper semantic relationships between the input and the rules. This can result in ineffective or redundant rules influencing the final decision.

To address this, we incorporate a Self-Attention-based rule-level attention mechanism that dynamically adjusts rule weights for each input sample, enabling the model to focus on the most informative rules.

Given an input feature vector $x\in R^{d}$ and R rules, calculating the firing strength by directly multiplying membership values in high-dimensional spaces often leads to numerical underflow.To address this, we compute the log-firing strength of the r-th rule as the initial activation. Derived from the Deformable GMF definition in Eq. (7), the log-activation is formulated as:


$$\begin{array}{c} ln(a_{r})=\sum_{d=\text{1}}^{D}-\frac{{\left(x_{d}-\left({\text{c}}_{d,r}+\Delta c_{d,r}\right)\right)}^{\text{2}}}{\text{2}\overset{\text{2}}{\underset{\text{d},\text{r}}{\sigma }}} \end{array}$$ (8)

Where ${\text{c}}_{d,r}$,$\Delta {\text{c}}_{d,r}$ and $\sigma_{d,r}$ are trainable parameters, correspond to the center, offset, and width parameters defined in Eq. (7), respectively.

To capture the complex dependencies within the feature space and dynamically assign importance to fuzzy rules, we employ a Multi-Head Self-Attention (MHSA) mechanism. Unlike simple additive attention, MHSA projects the input features into multiple subspaces, allowing the model to attend to different semantic aspects of the data simultaneously.

Given the input feature vector $x\in R^{d}$, we treat it as a sequence of length 1. The query (Q), key (K), and value (V) matrices are obtained via linear projections. The Multi-Head Attention is computed as follows:


$$\begin{array}{c} Head_{i}=Attention\left(x\overset{Q}{\underset{i}{W}},x\overset{K}{\underset{i}{W}},x\overset{V}{\underset{i}{W}}\right)\\ h\;=Concat\left(Head_{\text{1}},Head_{\text{2}},\ldots ,Head_{H}\right)W^{O} \end{array}$$ (9)

$$\begin{array}{c} s=hW_{fc}+b_{fc} \end{array}$$ (10)

$$\begin{array}{c} \overset{\text{att}}{\underset{\text{r}}{\alpha }}=\frac{exp\left(s_{r}\right)}{\sum_{j=\text{1}}^{R}exp\left(s_{j}\right)} \end{array}$$ (11)

Where $Head_{i}$ represents the output of the i-th attention head, H is the number of heads, and $W^{O}\in R^{DxD}$ is the output projection matrix. The vector $h\in R^{D}$ denotes the feature representation after self-attention. Subsequently, a linear transformation with weights $W_{fc}\in R^{DxR}$ and bias $b_{fc}\in R^{R}$ maps the attended features to the rule scores s. Finally, the rule attention weights $\alpha^{att}$ are obtained by applying the Softmax function.

### 3.4 Multi-view structure and fusion strategy

To effectively integrate information from multiple heterogeneous views, we propose a rule-activation-level fusion framework. Instead of concatenating multi-view features at the input layer, each view is processed by an independent fuzzy subsystem, which generates rule activation responses using Deformable GMFs and Self-Attention. The outputs from all views are then fused at the rule activation level, enabling cross-view information interaction without increasing input dimensionality.

Formally, let there be V views, with the input of view v denoted as $x^{\left(v\right)}\in R^{d_{v}}$, the fuzzy subsystem for view v produces:


$$\begin{array}{c} f^{\left(v\right)}=Softmax\left(a^{\left(v\right)}⊙s^{\left(v\right)}\right) \end{array}$$ (12)

Where $s^{\left(v\right)}\in R^{R}$ is the vector of raw rule activation strengths, $a^{\left(v\right)}\in R^{R}$ contains the rule attention weights from Self-Attention, ⊙ denotes element-wise multiplication, and R is the number of rules. The global fused activation vector is then computed as:


$$\begin{array}{c} f^{fused}=\sum_{v=\text{1}}^{V}\beta^{\left(v\right)}\cdot f^{\left(v\right)} \end{array}$$ (13)

Where $\beta^{\left(v\right)}$ represents the adaptive importance weight for view v. In our proposed framework, β is defined as a learnable parameter vector. To strictly satisfy the constraint $\sum_{v=\text{1}}^{V}\beta^{\left(v\right)}=\text{1}$ and ensure numerical stability, we apply a Softmax operation to the unnormalized weights during the forward pass. These weights are initialized uniformly and updated end-to-end alongside the antecedent and consequent parameters via the AdamW optimizer. This allows the model to automatically learn the optimal contribution of each view based on the training loss.

This fusion strategy supports fixed weighting, mean fusion, or a view-level attention mechanism to dynamically adjust inter-view contributions per sample. By performing fusion at the rule level, the model preserves modularity, improves adaptability to heterogeneous features, and offers stronger interpretability and structural control—particularly beneficial for handling multi-source data with inconsistent dimensionality and semantic diversity.

## 4. Experimental design and results analysis

### 4.1 Dataset overview

To objectively evaluate the proposed method, we conducted experiments on five real-world public datasets, each representing different application domains and feature characteristics. Specifically, four of these datasets (Dermatology, Forest Type, Caltech7, and Handwritten) were selected from the UCI Machine Learning Repository and standard public benchmarks, while the Epileptic EEG dataset was adopted from [16]. The details of these datasets are as follows:

1. Dermatology – A medically oriented dermatology dataset containing both histopathological and clinical perspectives, used for extracting features relevant to specialized diagnosis.
2. Forest Type – A remote sensing dataset derived from satellite imagery of forests, with features extracted from spectral bands and wavelength information.
3. Epileptic EEG – A dataset of electroencephalogram (EEG) signals from epilepsy patients. Features are extracted using Discrete Wavelet Transform (DWT) and Wavelet Packet Decomposition (WPD) [16]. (Ethical Statement: This study utilizes a publicly available, de-identified dataset originally published by Andrzejak et al. As the data contains no personally identifiable information and is in the public domain for research use, independent ethical approval or participant consent was not required for this secondary analysis.)
4. Caltech7 – A multi-view image dataset consisting of 1,474 images. Features are extracted from three perspectives: Gabor filters, CENTRIST descriptors, and wavelet texture analysis.
5. Handwritten – A widely used multi-view learning dataset containing 2,000 samples with six different views, often employed as a benchmark for multi-view classification tasks.

We evaluated our model on five public multi-view datasets (Caltech7, Handwritten Numerals, Dermatology, Forest Type, and EEG DWT-WAV). To ensure reproducibility and fair comparison, we utilized pre-extracted feature representations stored in.mat format (e.g., HOG/GIST/LBP for Caltech7, and DWT/WAV for EEG), eliminating the need to process raw signals. For data preprocessing, zero values within the feature matrices were first adjusted by adding a negligible constant (${\text{10}}^{-\text{8}}$) to prevent numerical instability. Subsequently, all features were normalized using either Min-Max or Standard scaling to align heterogeneous scales across different modalities. The exact feature dimensionalities and complete pipeline scripts are thoroughly documented in our public repository.

### 4.2 Experimental settings

The proposed MDA-TSK-FS was implemented in PyTorch and conducted on a workstation equipped with an NVIDIA RTX 4090D GPU. To ensure reproducibility, we fixed random seeds for all twenty independent runs. The datasets were split into 80% training and 20% testing using stratified sampling to maintain class balance consistent with the original distributions. Crucially, all preprocessing steps (e.g., normalization) were fitted solely on the training set to prevent data leakage; the derived statistics were then applied to the test set. We employed the AdamW optimizer with an initial learning rate of 0.0005 and a weight decay of 1e-8. The batch size was set to 8, and the maximum number of epochs was 256. To prevent overfitting, we applied EarlyStopping with a patience of 50 epochs. The number of fuzzy rules was set to 51.

In addition to classification accuracy, we calculated the entropy and normalized entropy of rule-level self-attention weights to assess the uniformity of rule utilization. Previous studies had shown that changes in attention entropy were closely related to training stability, and low attention entropy could lead to unstable or even divergent training; therefore, attention entropy was commonly used as an important indicator for monitoring the training status in models involving attention mechanisms [17]. Moreover, the number of effective rules was also counted to characterize the actual participation of rules in model decisions. For the deformable Gaussian membership functions, the mean $\mu_{c}=\frac{\text{1}}{N}\sum C_{i}$ and standard deviation $\sigma_{c}=\sqrt{\frac{\text{1}}{N}\sum {\left(C_{i}-\mu_{c}\right)}^{\text{2}}}$ were used to evaluate the stability of the Center, Sigma, and Offset parameters. These metrics provided quantitative support for the observations from the box plots, thereby assessing the stability and alignment of the deformable Gaussian antecedents across different datasets or views.

### 4.3 Results

To validate the effectiveness of the proposed MDA-TSK-FS model in multi-view data fusion tasks, we conducted comparative experiments against a set of existing representative TSK models, including the standard TSK-FS, MBGD-RDA [18], TSK-MBGD-UR, TSK-MBGD-BN [19], HTSK, HTSK-LN, HTSK-LN-RELU [20], as well as TSK variants under various multi-view fusion frameworks, namely DMCCA-TSK, DGCCA-TSK, DTCCA-TSK, DCCAEG-TSK [10], and MVD-TSK-FS [21]. All models were evaluated on five benchmark datasets, with classification accuracy used as the performance metric.

Table 1 presents the classification accuracies of all models across the five datasets. For the proposed MDA-TSK-FS, we conducted 20 independent runs and reported the results as “Mean±Standard Deviation” to reflect the stability and robustness of the model.For the baseline comparisons, we adopted the results reported in the recent study by Nie et al. [21], which provided a comprehensive evaluation of these TSK variants on the same datasets. Since the run-wise variance data (standard deviations) for these baselines were not available in the cited literature [21], we treated their reported mean accuracies as fixed benchmarks. To rigorously validate the improvements, we performed a one-sample t-test comparing the distribution of our 20 independent runs against the best result reported by the baselines. The asterisks (*) in Table 1 indicate that MDA-TSK-FS achieves a statistically significant improvement (p < 0.05) on all five datasets.

**Table 1:** Classification Accuracy of Different Models.

Model	Caltech7	Handwritten	Dermatology	Forest	EEG
**TSK-FS** ^**†**^	0.9153	0.9100	0.9075	0.8275	0.6050
**HTSK** ^**†**^	0.9390	0.9700	0.9600	0.8300	0.6750
**MBGD-RDA** ^**†**^	0.9356	0.9600	0.9700	0.8475	0.6650
**TSK-MBGD-UR** ^**†**^	0.9254	0.9600	0.9550	0.8818	0.5950
**TSK-MBGD-BN** ^**†**^	0.9254	0.9675	0.9324	0.8853	0.6000
**HTSK-LN** ^**†**^	0.9390	0.9500	0.9650	0.8324	0.6450
**HTSK-LN-RELU** ^**†**^	0.9322	0.9700	0.9600	0.8500	0.6550
**DMCCA-TSK** ^**†**^	0.9288	0.9100	0.9375	0.8514	0.4200
**DGCCA-TSK** ^**†**^	0.9390	0.8900	0.8650	0.8514	0.6150
**DTCCA-TSK** ^**†**^	0.9254	0.9400	0.8950	0.8243	0.5700
**DCCAEG-TSK** ^**†**^	0.9119	0.9300	0.9075	0.8485	0.6600
**MVD-TSK-FS** ^**†**^	0.9322	0.9100	0.9772	0.8581	0.5750
**MDA-TSK-FS**	0.9438 ± 0.0097*	0.9862 ± 0.0024*	0.9858 ± 0.0109*	0.8857 ± 0.0162*	0.6975 ± 0.0127*

**Note:** †The results for all baseline models are directly cited from the comprehensive benchmark study by Nie et al. [20], which reported mean accuracy only. The results of MDA-TSK-FS are reported as “Mean ± Standard Deviation” calculated over 20 independent runs. *indicates that the performance improvement of MDA-TSK-FS is statistically significant (p < 0.05) compared to the best-performing baseline, verified via a one-sample t-test.

**Fig 2:**
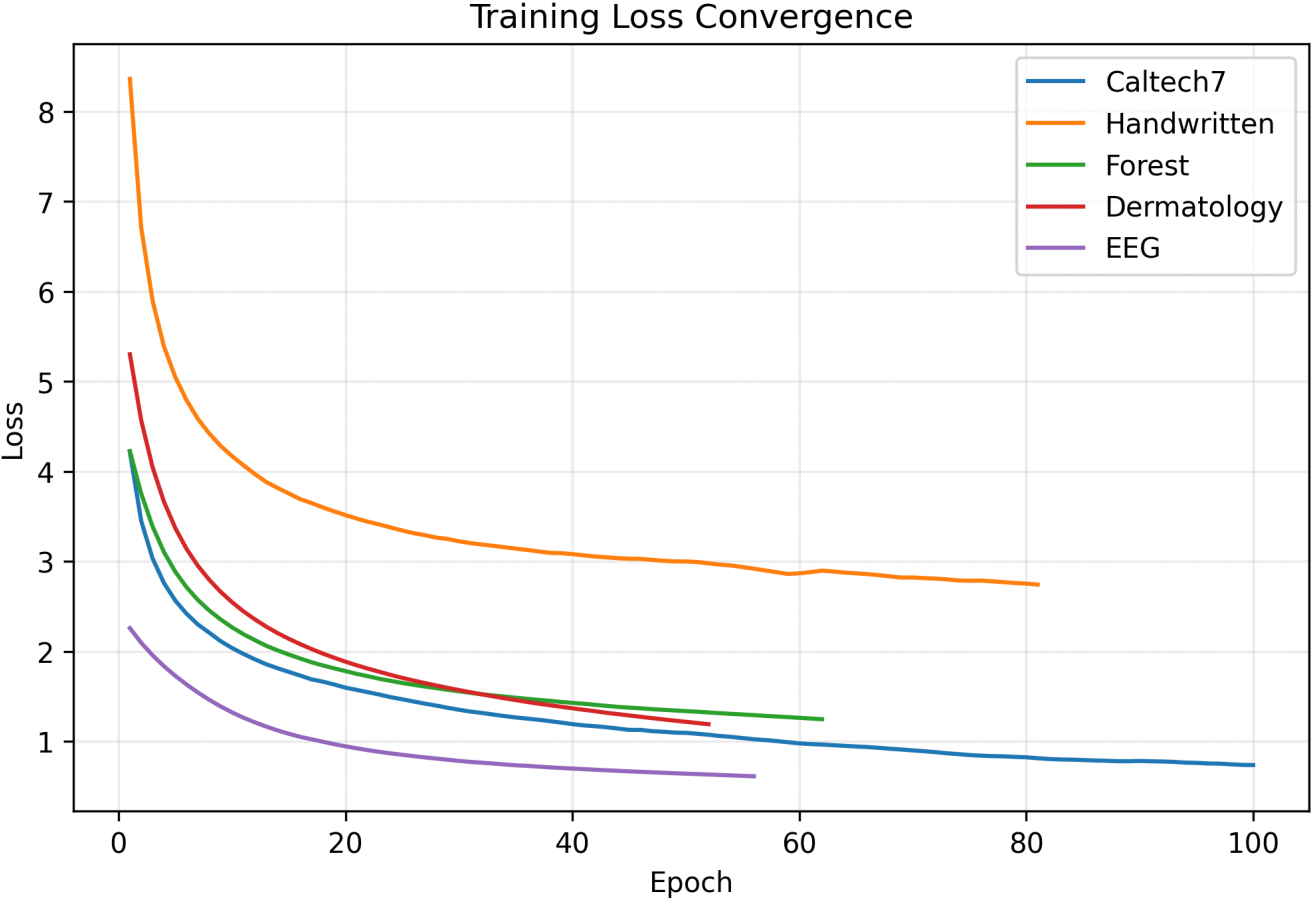
MDA-TSK-FS Convergence curves of all datasets.

The proposed MDA-TSK-FS consistently achieved the best or near-best performance in all cases, reaching accuracies of 0.9438, 0.9862, 0.9858, 0.8857, and 0.6975 on the Caltech7, Handwritten, Dermatology, Forest, and EEG datasets, respectively. Notably, MDA-TSK-FS demonstrated outstanding performance on the Handwritten and Dermatology datasets, significantly surpassing traditional TSK models (e.g., TSK-FS’s 0.9100 and 0.9075). Furthermore, on the Forest and EEG datasets, MDA-TSK-FS achieved 0.8857 and 0.6975, outperforming all competing models. In contrast, strong baselines such as TSK-MBGD-BN and HTSK-LN-RELU achieved only 0.6000 and 0.6550 on EEG, respectively, highlighting the effectiveness of integrating deformable Gaussian membership functions with an attention-based fusion mechanism in enhancing the model’s capacity to model complex, heterogeneous data distributions. As shown in Fig 2, we plotted the convergence curves of each dataset over the epochs. The loss decreased rapidly across all datasets, and convergence was generally achieved between 50 and 100 epochs.

**Fig 3:**
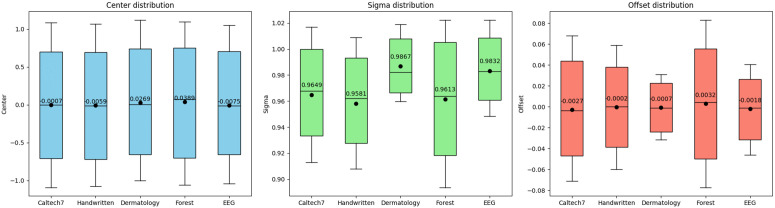
Distribution of rule parameters across datasets.

We plotted box plots of the rule parameter distributions for each dataset, as shown in Fig 3. The means of the Centers were generally close to zero and exhibited little variation across datasets, indicating that the deformable Gaussian functions could stably align the centers of multi-view features and prevent systematic shifts in rule distributions. Furthermore, the Sigmas remained within the range of 0.95–0.99 across different datasets, demonstrating consistent deformation in rule widths: even when facing datasets with significantly different distributions, the expansion and contraction of the rules stayed within a reasonable range, ensuring balanced feature coverage. The means of the Offsets were close to zero, suggesting that only minimal translations were required during parameter adjustment to adapt to the multi-view feature space.

**Fig 4:**
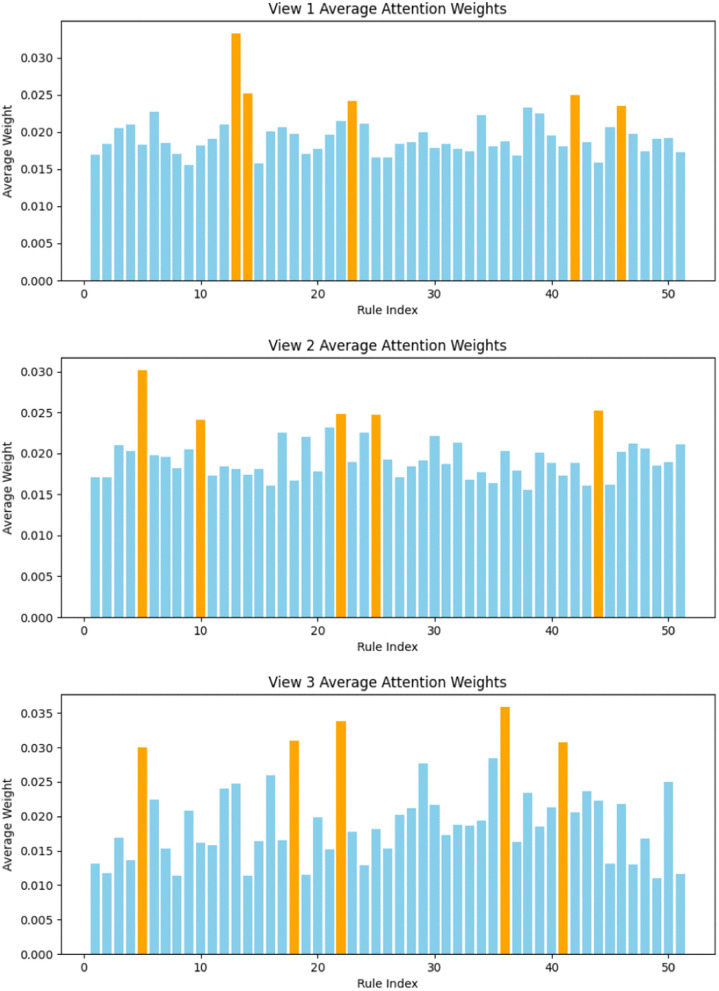
Distribution of rule attention weights across views in the Caltech7 dataset.

To further investigate the utilization of rules in the MDA-TSK-FS model under multi-view feature fusion, we computed the average rule attention weights for all 1,474 samples in the Caltech7 dataset and plotted the corresponding weight distributions for each view (Fig 4).

The attention distribution of the first view (48-dimensional features) was relatively uniform, with the Top-5 rules exhibiting significantly higher weights: Rule 13, Rule 14, Rule 23, Rule 24, and Rule 46. This indicates that the model primarily relied on these rules for discrimination in this modality, while other rules still contributed to a lesser extent. For the second view (40-dimensional features), the Top-5 rules were Rule 5, Rule 10, Rule 22, Rule 25, and Rule 44, showing clear differences from the rules emphasized in the first view, which reflects that discriminative information is distributed differently across modalities and that the model can flexibly select important rules according to different feature patterns. In the third view (254-dimensional features), the Top-5 rules were Rule 5, Rule 18, Rule 22, Rule 36, and Rule 41. Notably, Rule 5 and Rule 22 also exhibited high importance in the second view, suggesting that these two rules may represent cross-modal discriminative patterns. Overall, these qualitative results demonstrate that the MDA-TSK-FS model dynamically allocates importance weights to fuzzy rules via rule-level self-attention, reinforcing core rules while maintaining flexible adjustment of non-core rules, thereby achieving efficient and stable discrimination in multi-view fuzzy systems.

Next, to further understand the rule mechanisms underlying the model’s performance, we computed, for each view, the contribution rates of the Top-5 rules, entropy, normalized entropy, the number of effective rules, and the proportion of active rules (Table 2).

**Table 2:** Statistics of rule-level self-attention weights for each view.

View	Top 5	entropy	normalized entropy	effective rules	proportion
**Caltech7 View1**	13.12%	3.9207	0.9972	50.44	98.9%
**Caltech7 View2**	12.90%	3.9219	0.9975	50.5	99.01%
**Caltech7 View3**	16.15%	3.8849	0.9881	48.66	95.42%
**Handwritten View1**	15.461%	3.8996	0.9918	49.38	96.83%
**Handwritten View2**	12.698%	3.9241	0.9980	50.61	99.23%
**Handwritten View3**	22.041%	3.8222	0.9721	45.71	89.62%
**Handwritten View4**	12.692%	3.9252	0.9983	50.66	99.34%
**Handwritten View5**	9.881%	3.9252	0.9983	50.66	99.34%
**Handwritten View6**	14.561%	3.8956	0.9908	49.18	96.44%
**Dermatology View1**	13.958%	3.9042	0.9930	49.61	97.28%
**Dermatology View2**	12.145%	3.9222	0.9976	50.51	99.05%
**Forest View1**	16.792%	3.8716	0.9847	48.02	94.15%
**Forest View2**	14.059%	3.9001	0.9919	49.41	96.88%
**EEG View1**	12.845%	3.9188	0.9967	50.34	98.71%
**EEG View2**	16.132%	3.8629	0.9825	47.61	93.34%

The results showed that the normalized entropy of most views was close to 1, indicating that the majority of rules contributed meaningfully to the model’s decisions. A few views, such as Handwritten View 3 and EEG View 2, exhibited slightly lower normalized entropy, suggesting a more concentrated rule distribution, where a small number of rules carried greater decision weight. The contribution rates of the Top-5 rules remained around 12%–16% for most views, whereas Handwritten View 3 reached 22%, indicating that decisions in certain views relied more heavily on a few core rules. Conversely, views with lower Top-5 contribution rates, such as Handwritten View 5 (9.88%), showed a more balanced distribution of rule importance. Regarding the number of effective rules, most views approached the total number of rules, with the proportion of active rules exceeding 95%, while a few views (e.g., Handwritten View 3 and EEG View 2) had slightly fewer effective rules, consistent with their higher Top-5 contribution rates. These statistics clearly illustrate the role of rule-level self-attention in the MDA-TSK-FS model: the Top-5 contribution rate reflects the static importance distribution of rules across a view, whereas rule-level self-attention weights are dynamically assigned for each sample, enabling the model to flexibly select and emphasize rules based on input. In views with high Top-5 contribution rates, a few core rules are more likely to receive higher attention weights, dominating the model’s decisions; in views with lower Top-5 contribution rates, attention weights are more evenly distributed, reducing dependency on specific rules and enhancing robustness to complex or noisy samples.

To demonstrate human-interpretable semantics, the learned rules can be explicitly extracted. For instance, the highly attended Rule 13 in View 1 of Caltech7 (Fig 4) is expressed as:


$$\begin{array}{c} \begin{array}{c} IF{\;x}_{\text{1}\;}is{\;A}_{\text{1},\text{1}\text{3}\;}\left(centered\;at{\;c}_{\text{1},\text{1}\text{3}}+\Delta c_{\text{1},\text{13}}\right)\\ AND\;x_{\text{2}}\;is\;A_{\text{2},\text{1}\text{3}\;}\left(centeredat{\;c}_{\text{2},\text{1}\text{3}}+\Delta c_{\text{2},\text{13}}\right)\\ THEN\;y_{\text{1}\text{3}}=\sum_{d=\text{1}}^{D}a_{d,\text{1}\text{3}\;}x_{d}+b_{\text{1}\text{3}} \end{array} \end{array}$$ (14)

The attention weights (α) directly reflect view relevance; as shown in Fig 4, View 1 prioritizes texture-specific rules (Rules 13, 23), while View 3 selects higher-level semantic rules (Rules 36, 41). Furthermore, compared to standard TSK baselines (e.g., our MDA-Basic) which suffer from uniform rule firing and overlap, MDA-TSK-FS enforces rule sparsity. As indicated in Table 2, the attention mechanism concentrates the decision weight on a compact subset of “effective rules” (the Top-5 rules), significantly reducing redundancy and mitigating rule explosion.

### 4.4 Ablation study

To further investigate the contribution of each key component in the MDA-TSK-FS model, we conducted a systematic ablation study by selectively removing or retaining the deformable Gaussian antecedent and the rule-level attention mechanism. Four model variants were constructed for comparison: MDA-Basic, the baseline version using standard Gaussian membership functions without any enhanced structures, served as the lower-bound reference. MDA(w/o Attn) augmented the baseline with deformable Gaussian antecedents, allowing each rule center to adaptively shift, but excluded the attention mechanism to evaluate the independent effect of the D module. MDA(w/o D) maintained static Gaussian antecedents while introducing the rule-level attention mechanism, where a multi-head attention network dynamically adjusted rule activation weights, thus assessing the attention mechanism’s effectiveness in the absence of deformable antecedents. Finally, the complete MDA-TSK-FS model integrated both deformable Gaussian antecedents and rule-level attention, combining antecedent flexibility with adaptive rule selection.

The experimental results, as shown in Table 3, indicate that MDA-TSK-FS achieved the highest accuracy across all five datasets, confirming the synergistic advantage of combining the two modules. Incorporating only the deformable antecedent structure, MDA(w/o Attn) demonstrated substantial gains over MDA-Basic on datasets such as Dermatology and EEG, showing that learnable center shifts significantly enhance the regional representational capacity of rules. Similarly, MDA(w/o D) consistently outperformed MDA-Basic, indicating that the rule-level attention mechanism effectively adjusts rule importance according to input, thereby improving discrimination capability. On the EEG dataset in particular, the deformable antecedent and attention mechanism individually improved accuracy by approximately 7% and 6%, respectively, and their combination further boosted performance to 69.75%. These results clearly demonstrate that both components are individually effective, and when combined, they offer stronger complementarity and improved generalization ability.

**Table 3:** Ablation experiment results of MDA-TSK-FS.

Model	Caltech7	Handwritten	Dermatology	Forest	EEG
**MDA-Basic**	0.9211 ± 0.0078	0.9784 ± 0.0045	0.9580 ± 0.0067	0.8556 ± 0.0092	0.6050 ± 0.0145
**MDA(w/o Attn)**	0.9208 ± 0.0082	0.9768 ± 0.0051	0.9694 ± 0.0059	0.8551 ± 0.0088	0.6750 ± 0.0145
**MDA(w/o D)**	0.9356 ± 0.0082	0.9862 ± 0.0051	0.9794 ± 0.0059	0.8642 ± 0.0088	0.6650 ± 0.0138
**MDA-TSK-FS**	**0.9438 ± 0.0056**	**0.9864 ± 0.0032**	**0.9858 ± 0.0028**	**0.8857 ± 0.0089**	**0.6975 ± 0.0123**

### 4.5 Computational complexity analysis

Addressing the potential concern of “rule explosion” in high-dimensional multi-view fuzzy systems, we evaluated the practical efficiency of the proposed MDA-TSK-FS. Although the introduction of the multi-head attention mechanism and deformable parameters increases the model’s complexity compared to standard zero-order TSK systems, the computational cost remains highly manageable for practical deployment.

We conducted inference speed tests on the Caltech7 dataset (which contains high-dimensional views up to 254 dimensions) using an NVIDIA RTX 4090D GPU. The model contains approximately 0.47 M trainable parameters. The average inference time is approximately 1.23 ms per sample, enabling real-time processing capabilities. Furthermore, as shown in Fig 2, the training process typically achieves convergence within 50–100 epochs, demonstrating high training efficiency. These results indicate that MDA-TSK-FS effectively balances high classification performance with computational feasibility.

## 5. Conclusion

This paper proposes MDA-TSK-FS, a multi-view TSK fuzzy system integrating deformable Gaussian antecedents and rule-level attention mechanisms. By dynamically adjusting membership function centers via learnable offsets and adaptively weighting rules with multi-head attention, the model enhances flexibility, expressiveness, and discrimination. Experiments on multiple benchmarks demonstrate superior accuracy, stability, and generalization compared to classical models. Ablation studies confirm the complementary benefits of deformable structures and attention. This work establishes a novel paradigm for multi-view fuzzy modeling, offering both strong interpretability and significant application potential. Future research may incorporate uncertainty modeling, graph structures, or advanced cross-modal fusion to address more complex tasks in fields such as clinical diagnosis and remote sensing.

## Supporting information

S1 File Supporting data archive. This compressed archive contains the underlying data for the experimental results, including loss data.xlsx (which records the training loss data across multiple datasets) and accuracy data.xlsx (which contains the detailed accuracy data from independent experimental runs).
(ZIP)

## References

[1] Zhao J, Xie X, Xu X, Sun S. Multi-view learning overview: Recent progress and new challenges. Information Fusion. 2017;38:43–54. doi: 10.1016/j.inffus.2017.02.007

[2] Takagi T, Sugeno M. Fuzzy identification of systems and its applications to modeling and control. IEEE Trans Syst, Man, Cybern. 1985;SMC-15(1):116–32. doi: 10.1109/tsmc.1985.6313399

[3] Cui Y, Wu D, Xu Y, editors. Curse of dimensionality for TSK fuzzy neural networks: Explanation and solutions. 2021 International Joint Conference on Neural Networks (IJCNN); 2021: IEEE.

[4] Mendel JM. Uncertain rule-based fuzzy systems. Introduction and new directions. 2017;684.

[5] Tang Z, editor Multi-view clustering: a brief review. 2018 3rd International Conference on Robotics and Automation Engineering (ICRAE); 2018: IEEE.

[6] Park G, Mersereau R, Smith MJT. Initialization of deformable templates using weighted Gaussian approximations. In: 2000 IEEE International Conference on Acoustics, Speech, and Signal Processing. Proceedings (Cat. No.00CH37100). 2231–4. doi: 10.1109/icassp.2000.859282

[7] Liu S, Oh S-K, Pedrycz W, Yang B, Wang L, Yoon JH. Reinforced Interval Type-2 Fuzzy Clustering-Based Neural Network Realized Through Attention-Based Clustering Mechanism and Successive Learning. IEEE Trans Fuzzy Syst. 2024;32(3):1208–22. doi: 10.1109/tfuzz.2023.3321197

[8] Zhou E, Chung FL, Wang S. Multiview fully interpretable TSK fuzzy classifier enhanced by multiview accompanying GMMs. IEEE Transactions on Fuzzy Systems. 2024;32(11):6288–302.

[9] Hardoon DR, Szedmak S, Shawe-Taylor J. Canonical correlation analysis: an overview with application to learning methods. Neural Comput. 2004;16(12):2639–64. doi: 10.1162/0899766042321814 15516276

[10] Wong HS, Wang L, Chan R, Zeng T. Deep Tensor CCA for Multi-view Learning. IEEE Trans Big Data. 2021;1–1. doi: 10.1109/tbdata.2021.3079234

[11] Peng Y, Xie W, Li J, Wu S, Wang Z, Hu B, et al., editors. LDG: Lightweight Deformable 3D Gaussians for Single-View Dynamic Scene Reconstruction. ICASSP 2025-2025 IEEE International Conference on Acoustics, Speech and Signal Processing (ICASSP); 2025: IEEE.

[12] Dai J, Qi H, Xiong Y, Li Y, Zhang G, Hu H, et al., editors. Deformable convolutional networks. Proceedings of the IEEE international conference on computer vision; 2017.

[13] Vaswani A, Shazeer N, Parmar N, Uszkoreit J, Jones L, Gomez AN. Attention is all you need. Advances in neural information processing systems. 2017;30.

[14] Wen Y, Chen S, Shrestha AK, editors. Fast Vision Transformer via Additive Attention. 2024 IEEE Conference on Artificial Intelligence (CAI); 2024: IEEE.

[15] Sahoo KK, Hazra R, Ijaz MF, Kim S, Singh PK, Mahmud M. MIC_FuzzyNET: Fuzzy Integral Based Ensemble for Automatic Classification of Musical Instruments From Audio Signals. IEEE Access. 2022;10:100797–811. doi: 10.1109/access.2022.3208126

[16] Zhang W, Deng Z, Wang J, Choi K-S, Zhang T, Luo X, et al. Transductive Multiview Modeling With Interpretable Rules, Matrix Factorization, and Cooperative Learning. IEEE Trans Cybern. 2022;52(10):11226–39. doi: 10.1109/TCYB.2021.3071451 34043519

[17] Zhai S, Likhomanenko T, Littwin E, Busbridge D, Ramapuram J, Zhang Y. Stabilizing transformer training by preventing attention entropy collapse. In: 2023.

[18] Wu D, Yuan Y, Huang J, Tan Y. Optimize TSK Fuzzy Systems for Regression Problems: Minibatch Gradient Descent With Regularization, DropRule, and AdaBound (MBGD-RDA). IEEE Trans Fuzzy Syst. 2020;28(5):1003–15. doi: 10.1109/tfuzz.2019.2958559

[19] Cui Y, Wu D, Huang J. Optimize TSK Fuzzy Systems for Classification Problems: Minibatch Gradient Descent With Uniform Regularization and Batch Normalization. IEEE Trans Fuzzy Syst. 2020;28(12):3065–75. doi: 10.1109/tfuzz.2020.2967282

[20] Cui Y, Xu Y, Peng R, Wu D. Layer Normalization for TSK Fuzzy System Optimization in Regression Problems. IEEE Trans Fuzzy Syst. 2023;31(1):254–64. doi: 10.1109/tfuzz.2022.3185464

[21] Nie L, Qian Z, Zhao Y, Jiang Y. A novel algorithm to multi-view TSK classification based on the Dirichlet distribution. In: 2023.

